# Supplementary material for: Resolving the full spectrum of human genome variation using Linked-Reads
Source: Genome Res. 2019 Apr;29(4):635–45. doi: 10.1101/gr.234443.118 (PMC6442396; doi:10.1101/gr.234443.118)
Supplement: Supplemental Material [file supp_gr.234443.118_Supplemental_longranger-2.2.zip › longranger-2.2.tar.bz2!/lr2.2-public/lib/stan/cmdstan/stan/doc/api/html/namespacestd.html]

Stan: std Namespace Reference


|  |  |
| --- | --- |
|  | Stan  2.5.0  probability, sampling & optimization |


- Main Page
- Related Pages
- Namespaces
- Classes
- Files

- Namespace List
- Namespace Members

All Classes Namespaces Files Functions Variables Typedefs Enumerations Enumerator Friends Macros Pages

Classes |
Functions

std Namespace Reference

Template specification of functions in std for Stan.
More...

|  |  |
| --- | --- |
| Classes | |
| struct | numeric\_limits< stan::agrad::fvar< T > > |
|  | |
| struct | numeric\_limits< stan::agrad::var > |
|  | Specialization of numeric limits for var objects. More... |
|  | |

|  |  |
| --- | --- |
| Functions | |
| int | isnan (const stan::agrad::var &a) |
|  | Checks if the given number is NaN. More... |
|  | |
| int | isinf (const stan::agrad::var &a) |
|  | Checks if the given number is infinite. More... |
|  | |

## Detailed Description

Template specification of functions in std for Stan.

## Function Documentation

|  |  |  |  |  |  |
| --- | --- | --- | --- | --- | --- |
| int std::isinf | ( | const stan::agrad::var & | *a* | ) |  |

inline

Checks if the given number is infinite.

Return `true` if the value of the a is positive or negative infinity.

Parameters
:   |  |  |
    | --- | --- |
    | a | Variable to test. |

Returns
:   `true` if value is infinite.

Definition at line 75 of file numeric\_limits.hpp.

|  |  |  |  |  |  |
| --- | --- | --- | --- | --- | --- |
| int std::isnan | ( | const stan::agrad::var & | *a* | ) |  |

inline

Checks if the given number is NaN.

Return `true` if the value of the specified variable is not a number.

Parameters
:   |  |  |
    | --- | --- |
    | a | Variable to test. |

Returns
:   `true` if value is not a number.

Definition at line 62 of file numeric\_limits.hpp.


---

[ Stan Home Page ]

*© 2011–2014,
Stan Development Team.*
